# Experimental Setup for Dynamic Analysis of Micro- and Nano-Mechanical Systems in Vacuum, Gas, and Liquid

**DOI:** 10.3390/mi10030162

**Published:** 2019-02-26

**Authors:** Bram van den Brink, Farbod Alijani, Murali Krishna Ghatkesar

**Affiliations:** Department of Precision and Microsystems Engineering, Delft University of Technology, Mekelweg 2, 2628CD Delft, The Netherlands

**Keywords:** modal testing, experimental modal analysis, microsystems, micro-cantilevers, liquid, fluid, vacuum, dynamics, vibration analysis, AFM, Atomic Force Microscopy, vibration

## Abstract

An experimental setup to perform dynamic analysis of a micro- and nano-mechanical system in vacuum, gas, and liquid is presented. The setup mainly consists of a piezoelectric excitation part and the chamber that can be either evacuated for vacuum, or filled with gas or water. The design of the piezoelectric actuator was based on a Langevin transducer. The chamber is made out of materials that can sustain: vacuum, variety of gases and different types of liquids (mild acids, alkalies, common alcohols and oils). All the experiments were performed on commercial cantilevers used for contact and tapping mode Atomic Force Microscopy (AFM) with stiffness 0.2 N/m and 48 N/m, respectively, in vacuum, air and water. The performance of the setup was evaluated by comparing the measured actuator response to a finite element model. The frequency responses of the two AFM cantilevers measured were compared to analytical equations. A vacuum level of 0.6 mbar was obtained. The setup has a bandwidth of 10–550 kHz in vacuum and air, and a bandwidth of 50–550 kHz in liquid. The dynamic responses of the cantilevers show good agreement with theory in all media.

## 1. Introduction

Micro- and nano-mechanical resonators have emerged as useful tools for diverse sensing applications [1,2,3,4,5,6]. These sensors operate by measuring the change in dynamical behavior of a resonating structure, caused by the quantity of interest. Among many resonating structures, micro- and nano-cantilevers have emerged as the most popular structures for sensing applications. They are being used as sensors in various environments including vacuum, gas and liquid. There is much literature on the sensing functionality of a resonator in a particular environment, however, there is little to no information on how to build a setup for dynamic analysis of these tiny devices that allows changing the environment around the device to vacuum, gas or liquid without having to exchange the device. Such a setup is useful in applications such as hydrogels [7,8,9,10] (highly absorbant polymer network) to quantitatively characterize their absolute absorption capability from dry to fully absorbed state. This multi-environment characterization could also be useful to characterize zeolites [11,12,13,14] (nanoporous adsorbant mineral). By changing the environment from vacuum to different fluids and monitoring the material at different stages of adsorption, their absolute uptake capacity can be quantified.

When the resonator is operated in fluid, damping due to the fluid itself has a considerable effect on the dynamics of the system [15,16]. These effects have to be taken into account to correctly identify the quantity of interest. Several analytical models have been proposed [17,18,19,20,21,22] based on the Navier–Stokes equations, and experimentally verified, to describe the effects of the fluidic added mass and the damping related to the drag of the structure in fluid. To achieve a higher accuracy and to enable the modeling of more complex structures, the fluid–structure interaction of cantilever beams [23] and plates [24] was investigated using finite element models. Both models encapsulate the structure in a three-dimensional sphere of Navier–Stokes elements describing an incompressible viscous fluid, which is located in an infinite inviscid potential flow domain. The dynamic characteristics obtained from these models are based on certain assumptions of boundary conditions, dimensions, material properties, and damping present in the system. In practice, these parameters deviate due to the fabrication process of the materials and components, or change during the lifetime of the system [25]. Therefore, the need to characterize system dynamics in practice is of importance.

To perform dynamic analysis, the device needs to be excited. Piezoelectric base excitation is often employed to perform modal testing of microsystem in vacuum or gaseous environments [26]. A microsystem is attached either directly or by a holder to a piezoelectric element. In this way, the base of the microsystem is excited, causing vibrations in the structure. The advantage of this technique is that the microsystem in question does not have to be altered in any way. In addition, the effect of the actuator on the dynamics of the system is negligible in vacuum and gaseous environments.

Difficulties with this excitation method arise when applied in liquid environments in the form of spurious peaks, also known as “the forest of peaks” [27]. These peaks interfere with the dynamics of the cantilever itself, hindering quality factor and resonance frequency identification. Another concern is fluid-borne excitation of the microsystem by the vibration of the microsystem holder in the liquid, causing acoustic waves in the liquid [28], of which the forcing on the structure is difficult to model.

Efforts have been made to understand and reduce these spurious peaks, which some researchers contributed to the design of the liquid environment [27,29,30], and others to the microsystem holder [31,32,33].

We developed a setup to experimentally obtain the vibration response of micro- and nano-mechanical systems in vacuum, gas, and liquid. Here, the design of the setup is discussed, and its performance is evaluated by comparing the actual actuator response to a Finite Element Analysis (FEA) model. In addition, the frequency response of two Atomic Force Microscopy (AFM) cantilevers measured in vacuum, air, and water are compared to analytical results.

## 2. Materials and Methods

### 2.1. Design

The design of a piezoelectric actuator for base excitation is similar to the design of an ultrasonic transducer [34]. Both use a piezoelectric element (piezo) to convert electrical power to mechanical vibrations and transmit these to a target body, i.e., the chip base. The application however, is slightly different. Emitting ultrasonic transducers are often designed to be used at a relatively narrow frequency band. For optimal performance, a resonance frequency of the transducer is chosen as operating frequency to maximize the vibration amplitude. The base actuator, however, has to operate in a wide range of frequencies to excite multiple resonance frequencies of the microsystem. The suspension of the actuator also differs. Ultrasonic transducers are suspended at a node of the operational mode of the actuator to prevent transmitting vibrations to the setup, and, if necessary, protecting the actuator from the (liquid) environment.

**Figure 1:**
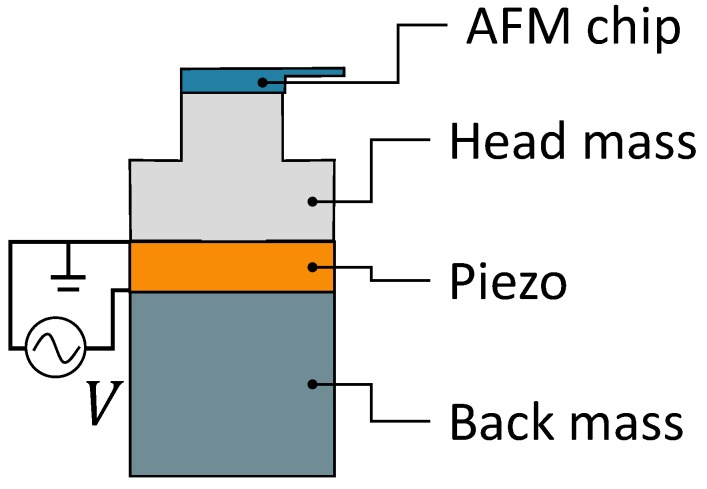
Schematic representation of the elementary components of a Langevin transducer, consisting of a head mass, a piezoelectric element, and a back mass.

Due to the desired large bandwidth, preferably in the kHz range, the actuator for microsystem base excitation is based on the design of a Langevin transducer [35,36]. A schematic of this design is shown in Figure 1. The transducer consists of the head mass, the piezoelectric element, and the back mass. The back mass acts as an inertial balance mass and will, in combination with the head mass, determine the amount of force which can be delivered to the target body. The first resonance frequency of the transducer is determined by the concatenation of all the components, which makes this design more fit for lower operating frequencies. The shape of the head mass depends on the application. Especially for power ultrasonics such as ultrasonic welding and ultrasonic cutting, the head mass is usually shaped in such a way that the force from the piezo is concentrated at the tip of the transducer. A shaped head mass is referred to as an acoustic horn. For the acoustic horn, Titanium was chosen as material because of its excellent chemical resistance. Stainless steel was chosen as back mass material due to its high characteristic impedance, which relates to the mass of the back mass scaled by its length at its first natural frequency. Polytetrafluoroethylene (PTFE) was chosen as the material for the environment chamber and chamber base, because of its excellent chemical resistance, and vacuum compatibility. In addition, the vibrations of the environment chamber will be damped by its relatively high material damping, which polymers generally possess.

**Figure 2:**
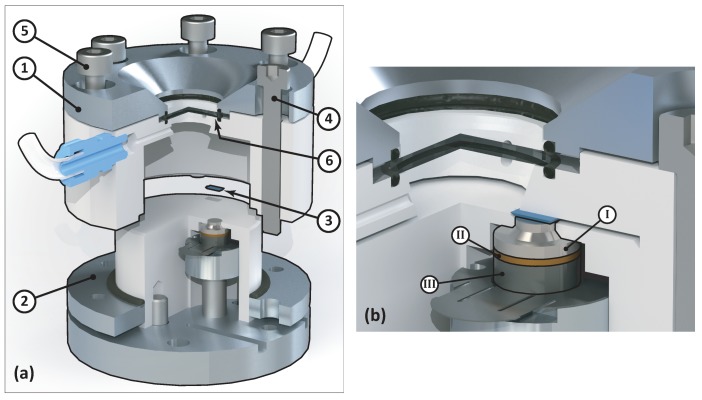
(**a**) Exploded cutaway view of the final design. The main parts of the design are: (1) the environment chamber top cap; (2) the environment chamber base; (3) the AFM chip; (4) the chip mounting bolts; (5) the chamber sealing bolts; and (6) the chamber window. (**b**) Cutaway view of the final design, zoomed at the actuator. The actuator consists of: (I) the acoustic horn; (II) the piezoelectric element; and (III) the back mass.

Figure 2a shows an exploded view of the setup. The environment chamber of the setup consists of a cap-shaped top (1), which can be placed over the environment base (2). The AFM chip (3) was clamped in between the environment chamber top and the base, hence integrating it in the chamber wall to minimize the amount of vibrations travelling from the actuator to the environment chamber. The clamping force was applied by tightening the two bolts (4), which are in line with the chip. These bolts go through holes in the lower flange and catch the thread in the base plate. The environment chamber was sealed by tightening the remaining four bolts (5), which catch the thread in the lower flange, pulling the cap-shaped top and lower flange together and compressing the O-ring seals onto the window.

The integration of the actuator is shown in Figure 2b. The acoustic horn (I) was integrated into a ring-shaped part of the environment chamber wall, which is reduced in thickness and acts as a suspension for the actuator assembly. The piezo (II) was electrically connected to the power source through a leaf spring, which was connected to the back mass (III), and through the acoustic horn, which was grounded. The leaf spring was of sufficiently low stiffness and ensures that no wires have to be attached to the moving parts. It is important to note that separating the piezo from the liquid is crucial in the design of a piezoelectric actuator. This introduces an extra component compared to designs aimed at gaseous environments or vacuum, where short-circuit protection is of less importance, due to the high electrical breakdown voltage of gasses compared to liquids. The introduction of this component and the way it was integrated in the environment chamber increases the complexity of the design.

**Figure 3:**
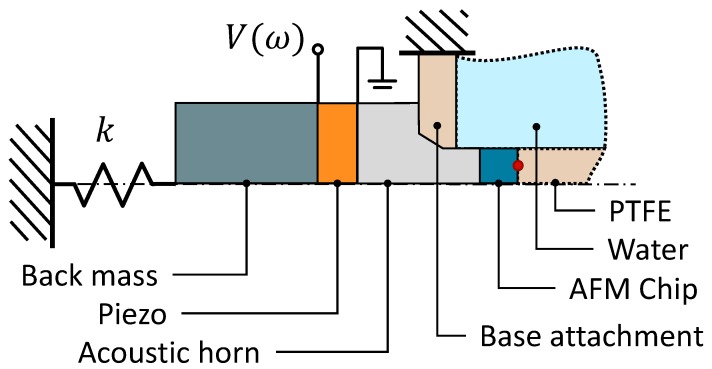
Axisymmetric view of the two-dimensional finite element model. The interface between the AFM chip and the PTFE, denoted by the red dot, was the point where the response of the chip base was calculated. The acoustic and solid mechanic domains are denoted by dotted lines and solid lines, respectively.

To evaluate the performance of the design, the vibration response of the actuator was modeled. After fabrication, the actual response of the chip base can be measured and compared to check the validity of the model. To this end, a 2D axisymmetric Acoustic-Piezoelectric frequency domain finite element analysis was performed, using the multi-physics Finite Element Analysis (FEA) interface of COMSOL. The setup of the model is shown in Figure 3 and can be divided into the pressure acoustics domain, the solid mechanics domain, and the electrostatics domain. The pressure acoustics domain is denoted by the dotted edges. In this domain, only compressional waves are present. A perfect acoustic match was set on the outer edges of the acoustic domain to ensure there are no resonant frequencies associated with these domains, assuming vibrations entering the liquid and the PTFE environment chamber are completely damped out. The acoustic domain was coupled with the solid mechanics domain, which is denoted by solid lines, at the interfacing edges. The solid mechanics domain considers, in addition to compressional waves, shear waves and the Poisson’s effect. The solid mechanics domain is coupled to the electrostatic domain by the piezoelectric material of the piezoelectric element. A voltage of 8 V was applied to the piezo in the electrostatic domain. This voltage was also used when the performance of the fabricated actuator was evaluated. The leaf spring was modeled as a spring *k*, distributed along the back end of the back mass. The suspension was fixed to the chamber base assuming the chamber base to be infinitely stiff. The response of the actuator was calculated at the cantilever base by taking the average absolute particle displacement parallel to the axis of symmetry at the front of the AFM chip.

### 2.2. Fabrication

**Figure 4:**
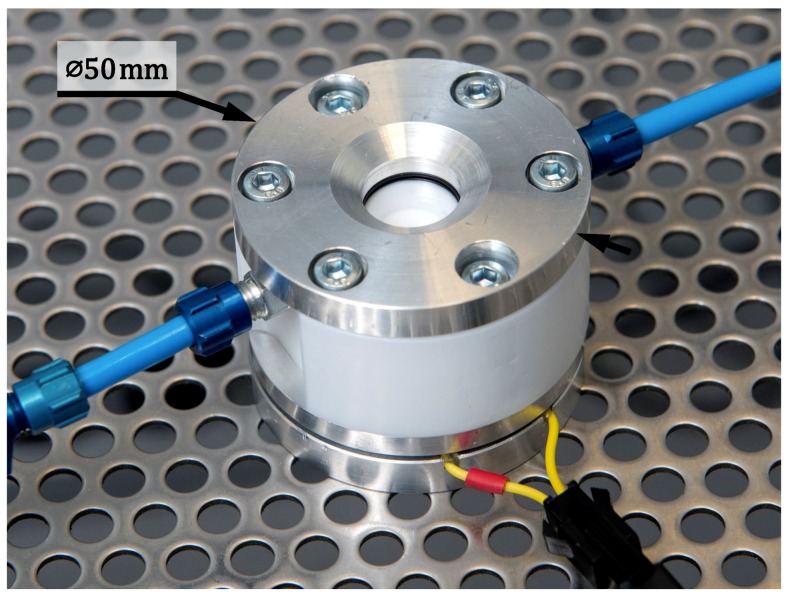
Setup after fabrication and assembly. The vacuum and liquid tubing can be connected using the blue connectors on each side of the setup. The electric wiring for the actuator is fed in through the base of the environment chamber.

Figure 4 shows the completed setup after fabrication and assembly. Most of these components were manufactured using a conventional lathe and mill; the leaf spring however, was laser cut. PTFE was found to be a material difficult to machine because of its poor thermal conduction and high creep rate. Therefore, it is advised to choose a different material in the future. On each respective side of the setup, a connector is present for tubing to connect liquid and vacuum components to the environment chamber, which has a volume of 0.34 mL. The electric wiring for the actuator is fed in through the base of the environment chamber. The electrical connection at the acoustic horn is made by carefully dispensing a droplet of conductive paint against the acoustic horn, while preventing the droplet from touching the back face of the piezo, which would short circuit the piezo. Subsequently, the wire is dipped in the droplet of conductive paint and fixed with super glue. The piezo, back mass, leaf spring, and the leaf spring mount were assembled with conductive epoxy, to which the live wire is connected.

### 2.3. Experimental Setup

To validate the design and to investigate the response of micro-cantilevers immersed in fluid, the base motion of the actuator was measured and the response of different AFM cantilevers was obtained in air, vacuum, and water. The cantilever vibrations were measured with a laser Doppler vibrometer (LDV), for which an optical window was incorporated into the top of the environment chamber.

**Figure 5:**
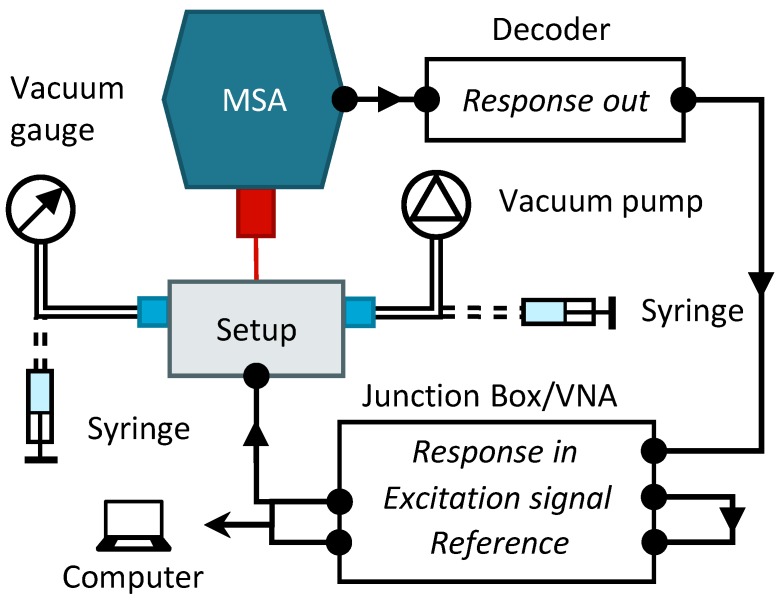
Schematic of experimental setup and auxiliary components. The arrows indicate the data acquisition path. The dotted tubing indicates the option of connecting either the vacuum components or the syringes for liquid exchange.

Figure 5 shows a schematic of the setup and its auxiliary components. The micro-cantilever was mounted in the setup and its vibrations were measured with a Micro-System Analyzer (MSA) LDV from Polytec Inc., of which the signal was fed into the decoder. The decoder translates the signal measured at the photo-detector of the MSA, which contains displacement and velocity data, into an electrical signal. This signal was fed into the MSA junction box or the Vector Network Analyzer (VNA). Both also host the signal generator that provides the excitation voltage for the actuator. The measured response from the LDV and the reference signal (i.e., the excitation signal) was fed into computer for analysis. In contrast to the junction box, which does a fast Fourier transform (FFT) of the signal, the VNA uses a homodyne detection scheme, i.e., it will only detect the response at the frequency of excitation.

The medium in the environment chamber can be exchanged by the in- and outlet ports on the setup (Festo 4 mm tubing connector). For the vacuum measurements, a Pfeiffer vacuum gauge was attached to one end of the environment chamber and a Pfeiffer diaphragm pump, with an ultimate pressure of 0.5 mbar, to the other end. For the measurements in water a syringe was connected to either side of the environment chamber. One of the syringes was used to impose a negative pressure, while the other was used to inject liquid into the environment chamber. This procedure reduces bubble forming.

The response of two different micro-cantilevers was measured in vacuum, air, and water by exciting the cantilever with a frequency sweep. The two cantilevers are meant for different AFM operating modes and vary in dimensions and stiffness. The CONT cantilever is meant for contact mode AFM and the NCLR cantilever is meant for tapping mode AFM. The stiffness of contact-mode cantilevers is usually relatively low to keep the applied lateral forces low, i.e., to reduce sample damage [37]. The NCLR tapping mode AFM probe, with typical dimensions 225±5 μm, 38±5 μm, 7.0±0.5 μm (length, width, thickness) has the higher first natural frequency of the two, 190 kHz. The CONT contact code AFM probe, with typical dimensions 450±5 μm, 50±5 μm, 2.0±0.5 μm (length, width, thickness), has a relatively low natural frequency of 13 kHz. This cantilever also has a significantly higher surface area.

## 3. Results

**Figure 6:**
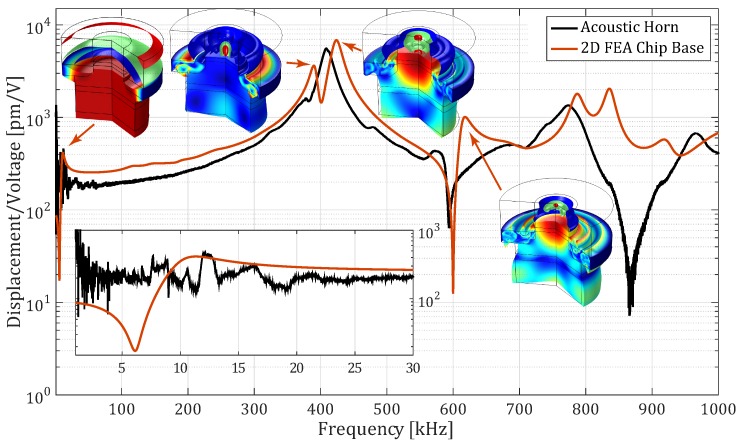
Measured frequency response of the actuator at the chip base compared to the FEA model evaluated at the top of the acoustic horn. In the bottom left corner, a zoom-in of the actuator response at low frequencies is shown.

The response of the acoustic horn was measured and compared to the response of the chip base from the FEA model. The response was measured at the top of the acoustic horn instead of the chip base, because the chip was clamped in-between the top and bottom of the environment chamber. The response at the chip base would be comparable though, due to the high first natural frequency of the chip base compared to the measurement bandwidth. The actuator was excited by a pseudo random excitation signal. As shown in Figure 6, the 2D FEA model gives a fair representation of the actuator response up to around 600 kHz. The first resonance frequency of the actuator is located at 377 kHz, which determines the bandwidth of the setup. This bandwidth can be extended up to around 550 kHz if the vibration responses from the micro-cantilevers are scaled by the response of the base, i.e., examining the transmissibility. The Operational Deflection Shape (ODS) at the third resonance frequency ( 617 kHz), however, curves the surface of the acoustic horn, introducing rotational effects on the base which cannot be compensated with the base response. Noise and small spurious peaks and drops are predominant in the region near the suspension mode of the FEA model and up to approximately 20 kHz, as shown in the zoomed in region of Figure 6. The excitation amplitude at the top of the acoustic horn has high variations in this region. This should be taken into consideration during the analysis of the cantilever response, because the energy fed into the system at those frequencies is irregular.

**Figure 7:**
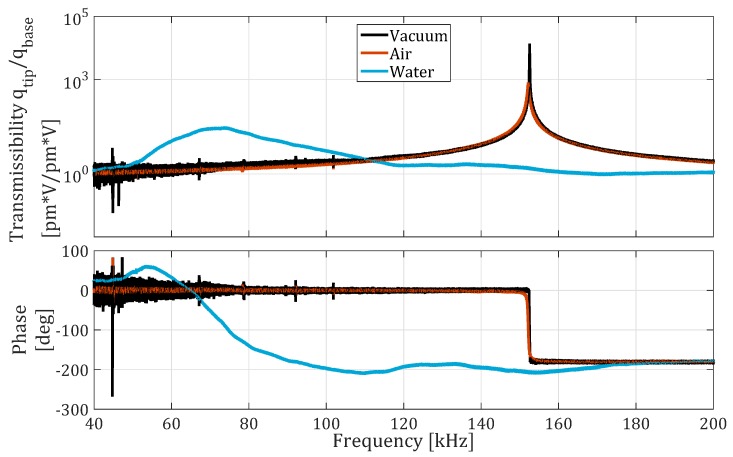
Frequency response of the NCLR cantilever, measured in in water, air, and vacuum ( 0.6 mbar).

Figure 7 shows the transmissibility of the NCLR cantilever in vacuum at 0.6 mbar, air, and water. The measured first resonance frequency of the cantilever in vacuum is 152.47 kHz. However, such a high deviation from the natural frequency specified by the manufacturer is not uncommon, and can be attributed to the fabrication process, in which tolerances of ±5 μm in length and width, and ±0.5 μm in thickness are typical.

Table 1 and Table 2 show the measured resonance frequency and the quality factor, identified by peak picking through the local fitting functionality of the Polytec MSA software. Next to the measured resonance frequencies and quality factors are their analytically calculated counterparts. The resonance frequency was calculated using the Pade approximation derived by Sader and Van Eysden [21], and the quality factor was calculated using Sader’s Method [19]. As expected, the resonance frequency in air is hardly affected by the presence of the medium. Its quality factor, however, drops from about 8000 to 500. Such a large drop is also observed in liquid, where the quality factor is about $\frac{1}{1000}$ of the value measured in vacuum and about $\frac{1}{100}$ of the value measured in air. The resonance frequency in water is about half the resonance frequency in vacuum and air. These values show good agreement with the estimated theoretical values taking into account that the aspect ratio of the cantilever surface can vary by 15% according to the supplier. The resonance frequency measured in vacuum was used to calculate the theoretical fluidic resonance frequency, therefore, assuming the uncertainty in fluid density to be negligible, the aspect ratio is the parameter with the greatest uncertainty influencing the theoretical resonance frequency. This uncertainty was reduced by varying the modeled cantilever dimensions within the tolerances given by the manufacturer until the theoretical resonance frequency in vacuum matches the measured resonance frequency in vacuum.

**Figure 8:**
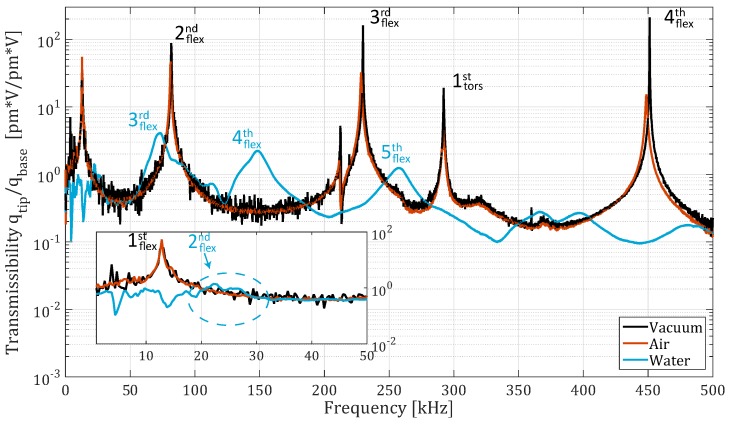
Frequency response of the CONT cantilever in water, air, and vacuum (1.3 mbar), measured with the vector network analyzer (VNA). The graph in the bottom left corner shows the response of the cantilever at low frequencies. The blue dashed ellipse surrounds the second resonance frequency in liquid.

Figure 8 shows the transmissibility of the cantilever in vacuum at 1.3 mbar, air, and water. It can be observed that the second resonance peak in water is quite irregular and does not have a clear peak. The first resonance peak in liquid is not even observable. In addition, the resonance peak of the first torsional ODS in liquid was not observed. Table 3 and Table 4 show the measured and theoretical resonance frequencies and quality factors. The torsional resonance was calculated with the Pade approximation derived by Sader and Van Eysden [21] and the quality factor of the torsional resonance was derived by using Green and Sader [20].

The measured response of the micro-cantilevers in liquid, identified by peak picking, approximately matches the theoretical values derived by Sader, Green, and Van Eysden [19,20,21], as observed in Table 1 and Table 4. The peaks however, are not symmetric around the resonance frequency. This was observed with all three of the cantilevers. This increases the uncertainty of the curve fit used to estimate the quality factors and natural frequencies.

The observed irregular peaks and the missing first resonance peak of the CONT cantilever in liquid have some similarities. They all arose while measuring in water, below approximately 50 kHz, and have a theoretical quality factor below 5.

Above 50 kHz, no notable spurious peaks were found in water, although some small local increases in amplitude can be observed. The amplitude of these peaks is much smaller however compared to the amplitude of the cantilever resonance peaks in water.

## 4. Discussion

During measurements, a vacuum level of 1.3 mbar–0.6 mbar was obtained. To get an indication of the significance of the effects of air at this pressure on the cantilever dynamics, the flow regime is calculated. The flow regime at this pressure can be estimated by evaluating the Knudsen number $Kn=\frac{\lambda }{L}$, where λ and *L* denote the mean free path and characteristic length scale respectively. The cantilever width is the characteristic length scale in the case of vibrating cantilevers [19], which is 50 μm for the largest measured cantilever (CONT). The mean free path at 1 mbar is approximately 100 μm, which results in a Knudsen number of 2, the transitional flow regime [38]. In this regime, the influence on the dynamics is not completely negligible, but lower then the influence of air at the continuum flow regime where added damping and added mass is invariant of pressure.

The setup bandwidth was found to be 10 kHz–550 kHz. The response of the setup within 10 kHz–20 kHz is irregular though, showing small spurious peaks and drops in amplitude. These could complicate measurements at this frequency range, especially in liquid due to the low quality factor of the modes. The second mode of the CONT cantilever has an irregular resonance peak. This might be caused by the actuator, which has a low excitation amplitude and an irregular response at lower frequencies. As a result, the modes might not be fed enough energy to build up, due to their high damping. Another reason could be that resonance peaks of the liquid are interfering with the resonance frequency of the cantilever modes. These peaks might stand out in the response of the the NCLR cantilever, which has no resonance frequencies of its own in this range. However, no resonances from the liquid were observed in the response of the NCLR cantilever at the frequency range in question. On the other hand, the cantilever is less susceptible to influences of resonances from the liquid, because of its smaller surface area and relatively high stiffness. Resonance peaks could successfully be measured in liquid from 50 kHz–500 kHz. It was observed, however, that the shape of these peaks in liquid are asymmetric. This might be due to a non-linear mechanism caused by the fluid–structure interaction. It was also found that the first resonance peaks of the flexural and the torsional mode of the CONT cantilever were not observed in the cantilever response in water. From the results in Table 3 and Table 4, it can be concluded that the resonance frequency of the first flexural mode is probably located outside the actuator bandwidth, where the amplitude of base excitation is rather low. In addition, the peak has a quality factor of 2 according to Sader’s model [19], which would make it extremely difficult to observe in the region with an irregular actuator response. The resonance frequency of the first torsional mode is located very close to that of the fourth flexural mode. Due to the high damping in liquid, the torsional mode might overlap with the third or fourth flexural mode. From the transmissibility (Figure 8) response of the CONT cantilever, a small peak at approximately 120 kHz can be observed. It was not possible to relate the ODS at this frequency to the first torsional mode, however, due to the large base excitation at this frequency compared to the amplitude of the torsional resonance. The ODS in addition will be a combination of the torsional mode and the third and/or fourth mode, which would require sophisticated MDOF identification techniques to confirm the exact location and quality factor of the torsional mode.

## 5. Conclusions

A versatile setup was designed with respect to sample selection, and operating environment. The setup allows vibration testing of cantilevers coated with a special material (hydrogel, zeolite, magnetostrictive, magnetic, etc.), as well as bare silicon cantilevers. The clamping mechanism also facilitates easy exchange of the microsystem chip. The environment chamber was built such that it can be used for measurements in: vacuum, different types of gasses, and different types of liquids including mild acids and alkalies, and common alcohols and oils. A vacuum level of 1.3 mbar–0.6 mbar was obtained. The setup has a bandwidth of 10 kHz–550 kHz. This range is large enough to excite up to four flexural modes of the cantilever in vacuum and air given the first natural frequency is chosen within 10 kHz–15 kHz.

## Figures and Tables

**Table 1:** Comparison between the experimentally measured and theoretical [21] fundamental frequency of the NCLR cantilever in different environments.

Mode	$f_{\mathrm{measured}}^{0.6\;\mathrm{mbar}}$	$f_{\mathrm{analytical}}^{\mathrm{vacuum}}$	$f_{\mathrm{measured}}^{\mathrm{air}}$	$f_{\mathrm{Sader}06}^{\mathrm{air}}$	$f_{\mathrm{measured}}^{\mathrm{water}}$	$f_{\mathrm{Sader}06}^{\mathrm{water}}$
	[kHz]	[kHz]	[kHz]	[kHz]	[kHz]	[kHz]
$1_{\mathrm{flex}}^{\mathrm{st}}$	152.47	152.76	152.07	152.27	72.10	85.51

**Table 2:** Comparison between the experimentally measured and theoretical [19] quality factor of the NCLR cantilever in different environments.

Mode	$Q_{\mathrm{measured}}^{0.6\;\mathrm{mbar}}$	$Q_{\mathrm{measured}}^{\mathrm{air}}$	$Q_{\mathrm{Sader}98}^{\mathrm{air}}$	$Q_{\mathrm{measured}}^{\mathrm{water}}$	$Q_{\mathrm{Sader}98}^{\mathrm{water}}$
$1_{\mathrm{flex}}^{\mathrm{st}}$	8214	497	680	7	9

**Table 3:** Comparison between the experimentally measured and theoretical [21] resonant frequencies of the CONT cantilever in different environments.

Mode	$f_{\mathrm{measured}}^{1.3\;\mathrm{mbar}}$	$f_{\mathrm{analytical}}^{\mathrm{vacuum}}$	$f_{\mathrm{measured}}^{\mathrm{air}}$	$f_{\mathrm{Sader}06}^{\mathrm{air}}$	$f_{\mathrm{measured}}^{\mathrm{water}}$	$f_{\mathrm{Sader}06}^{\mathrm{water}}$
	[kHz]	[kHz]	[kHz]	[kHz]	[kHz]	[kHz]
**Flex.**						
1st	12.98	13.01	12.80	12.92	-	4.25
2nd	81.80	81.55	81.22	81.41	25 ± 3	27.18
3rd	229.88	228.34	228.44	228.83	71.58	78.46
4th	451.40	447.46	448.89	449.49	148.71	158.98
5th	-	739.69	-	736.78	259.61	269.31
**Tors.**						
1st	292.26	293.77	292.06	291.76	-	149.18

**Table 4:** Comparison between the experimentally measured and the theoretical flexural [19], and torsional [20], quality factors of the CONT cantilever in different environments.

Mode	$Q_{\mathrm{measured}}^{1.3\;\mathrm{mbar}}$	$Q_{\mathrm{measured}}^{\mathrm{air}}$	$Q_{\mathrm{Sader}98}^{\mathrm{air}}$	$Q_{\mathrm{measured}}^{\mathrm{water}}$	$Q_{\mathrm{Sader}98}^{\mathrm{water}}$
**Flex.**					
1st	277	59	54	-	2
2nd	1638	168	154	3	5
3rd	4437	291	261	7	8
4th	5131	411	363	11	10
5th	-	-	460	12	13
**Tors.**					
1st	4519	558	1037	-	7
